# GPR84 regulates pulmonary inflammation by modulating neutrophil functions

**DOI:** 10.1038/s41401-023-01080-z

**Published:** 2023-04-04

**Authors:** Si-wei Wang, Qing Zhang, Dan Lu, You-chen Fang, Xiao-ci Yan, Jing Chen, Zhi-kan Xia, Qian-ting Yuan, Lin-hai Chen, Yang-ming Zhang, Fa-jun Nan, Xin Xie

**Affiliations:** 1 State Key Laboratory of Drug Research, National Center for Drug Screening, Shanghai Institute of Materia Medica, Chinese Academy of Sciences, Shanghai, 201203 China; 2 University of Chinese Academy of Sciences, Beijing, 100049 China; 3 School of Pharmaceutical Science and Technology, Hangzhou Institute for Advanced Study, University of Chinese Academy of Sciences, Hangzhou, 310024 China; 4 Shandong Laboratory of Yantai Drug Discovery, Bohai Rim Advanced Research Institute for Drug Discovery, Yantai, 264117 China; 5 School of Chinese Materia Medica, Nanjing University of Chinese Medicine, Nanjing, 210023 China; 6 Burgeon Therapeutics Co., Ltd., Shanghai, 201203 China

**Keywords:** GPR84, acute lung injury, inflammation, neutrophil, ROS, antagonist

## Abstract

Acute lung injury (ALI) is an acute, progressive hypoxic respiratory failure that could develop into acute respiratory distress syndrome (ARDS) with very high mortality rate. ALI is believed to be caused by uncontrolled inflammation, and multiple types of immune cells, especially neutrophils, are critically involved in the development of ALI. The treatment for ALI/ARDS is very limited, a better understanding of the pathogenesis and new therapies are urgently needed. Here we discover that GPR84, a medium chain fatty acid receptor, plays critical roles in ALI development by regulating neutrophil functions. GPR84 is highly upregulated in the cells isolated from the bronchoalveolar lavage fluid of LPS-induced ALI mice. GPR84 deficiency or blockage significantly ameliorated ALI mice lung inflammation by reducing neutrophils infiltration and oxidative stress. Further studies reveal that activation of GPR84 strongly induced reactive oxygen species production from neutrophils by stimulating Lyn, AKT and ERK1/2 activation and the assembly of the NADPH oxidase. These results reveal an important role of GPR84 in neutrophil functions and lung inflammation and strongly suggest that GPR84 is a potential drug target for ALI.

## Introduction

Acute lung injury (ALI) is characterized by acute respiratory insufficiency with tachypnea, severe hypoxemia defined by low arterial oxygen tension/inspired oxygen fraction ratio, and pulmonary edema with a mortality rate of around 40% and acute respiratory distress syndrome (ARDS) is the most severe form of ALI [1, 2]. ALI can be induced by lung infection, sepsis, local or systemic trauma. Severe Acute Respiratory Syndrome Coronavirus-2 (SARS-CoV-2, also called COVID-19) caused ARDS has resulted in more than 6 million deaths worldwide in less than 3 years [3]. Standard care for ALI/ARDS is very limited, and mechanical ventilation is the only treatment that reduces mortality [4]. A better understanding of the pathogenesis and new therapies are urgently needed.

According to the common etiology of ALI, direct lung injury or systemic inflammation-induced indirect lung jury induces pro-inflammatory polarization of the resting alveolar macrophages [5]. Following the release of proinflammatory cytokines and chemokines, massive neutrophils, monocytes and adaptive immune cells are recruited to damaged lung tissue and lead to uncontrolled lung inflammation [6]. Amongst those cells, neutrophils are the hallmark of ALI and the number of neutrophils in patient bronchoalveolar lavage fluid (BALF) is positively correlated with disease severity [7]. Activated neutrophils release numerous toxic substances, including reactive oxygen species (ROS), proinflammatory cytokines such as IL1β, IL6, and TNFα, granular enzymes, and neutrophil extracellular traps (NETs) [7]. ROS at low concentrations participate in signaling transduction and mediate pathogen defense, but unbalanced oxidative stress causes permanent damage [8]. In patients with severe ALI, ROS can even be detected in the expired air [9].

Considering the important role of neutrophils in the pathogenesis of ALI, targeting neutrophil function is a new way to develop ALI treatments. Inhibiting neutrophil migration and infiltration [10], reducing neutrophil-mediated oxidative stress [11], and promoting the clearance of NETs [12] have all been reported to provide protection in ALI. A number of G protein-coupled receptors (GPCRs) have been reported to be expressed by neutrophils and regulate neutrophil functions during ALI. For example, deletion or blockade of formyl peptide receptor 1 (FPR1) reduced neutrophil accumulation in lung [13] and oxidative stress [14] in ALI mice, leading to less damage of the lung tissue. C5aR is also an important GPCR that regulates neutrophil migration, oxidative stress and NETs production. Inhibition of C5aR significantly reduced the number of BALF neutrophils and ameliorates lung destruction in Influenza virus A (IAV) induced ALI [15]. Other GPCRs, including angiotensin receptor AT1 and LTB4 receptor, have also been suggested to influence ALI through neutrophils [16, 17].

GPR84 is a medium-chain fatty acids (MCFAs) receptor mainly expressed in neutrophils, monocytes, macrophages and other innate immune cells, indicating that GPR84 is closely associated with inflammation [18]. GPR84 has been reported to be upregulated by certain inflammatory stimuli, such as lipopolysaccharide (LPS) and TNFα [19]. Activation of GPR84 by MCFAs or synthetic agonists mediates neutrophil migration, macrophage phagocytosis, cytokine secretion and ROS production [18, 20]. GPR84 deficiency inhibits the production of inflammatory cytokines including IL1β, IL6, and TNFα in LPS-treated macrophages [21]. The multiple functions of GPR84 in immune system make it a rising target in inflammatory diseases such as ulcerative colitis and fibrotic diseases [22, 23].

Here we investigate the role of GPR84 in ALI and discover that deleting GPR84 or blocking the receptor with small molecule inhibitor all provide protective effects in ALI by reducing neutrophil recruitment, ROS production and degranulation. Further studies reveal that GPR84 promotes neutrophil degranulation and ROS production via Lyn, AKT and ERK1/2 pathways. These results indicate an important role of GPR84 in neutrophil functions and lung inflammation and suggest that GPR84 is a potential drug target for ALI.

## Materials and methods

### Mice

Male C57BL/B6 mice were purchased from the Shanghai Laboratory Animal Center (Shanghai, China). The generation and characterization of GPR84^−/−^ mice were described previously [22]. All mice were maintained under specific pathogen-free conditions in the animal facility of SIMM (Shanghai Institute of Materia Medica), with 12 h dark-light cycles and free access to food and water. Mice were used at 8–10 weeks of age. Animal experiments in this study were approved and conducted in accordance with the guidelines of the Institutional Animal Care and Use Committee of SIMM.

### LPS-induced ALI model

Mice were anesthetized and the intratracheally injected with 1 mg/kg LPS (L4391, Sigma-Aldrich, Darmstadt, Germany). For drug treatment, mice received vehicle, BGT-004 (25 and 50 mg/kg) 0.5 h before LPS instillation and were gavaged 12 h after induction. The dosage of BGT-004 was determined according to our previous report [24], where BGT-004 was referred to as compound **33**. Carboxymethylcellulose sodium (0.5%, *w*/*v*) was given as vehicle control. At 24 h after LPS instillation, the left lung lobe was collected from sacrificed mice, fixed in 4% (*w*/*v*) paraformaldehyde, embedded in paraffin, and sectioned for histological analysis and immunofluorescence staining. The histological grading of lung inflammation was carried out as described previously [25]. Other lobes were frozen at −80 °C for quantitative real-time (qRT)-PCR assay and enzyme-linked immunosorbent assay (ELISA). BALF was collected for analysis of immune cells by flow cytometry.

### Bleomycin-induced lung injury

Bleomycin (BLM, MB1039, Meilunbio, Dalian, China)-induced lung injury was performed as previously reported [26]. In brief, mice were anesthetized and 2 U/kg BLM was injected intratracheally. For drug treatment, mice orally received vehicle, BGT-004 (25 and 50 mg/kg) or pirfenidone (PFD, HY-B0673, MCE, Shanghai, China) twice daily. Mice were sacrificed 24 h or 7 days after BLM treatment. BALF was collected for analysis of immune cells by flow cytometry and lung tissues were reserved for histological analysis, immunofluorescence staining, and qRT-PCR assay.

### Isolation of bronchoalveolar lavage fluid (BALF) cells

BALF cells were collected by lavaging the lungs three times with 1 mL PBS buffer. The fluids were centrifuged at 400 × *g* for 10 min and the supernatant was assessed for total protein and pro-inflammatory cytokines. And cell pellets were harvested for flow cytometry analysis. Briefly, cells were resuspended in 1 mL of PBS buffer and stained with anti-CD45 PerCP (557235, BD Pharmingen, San Diego, CA, USA), anti-CD11b APC-Cy7 (A15390, Invitrogen, Carlsbad, CA, USA), anti-Ly6G PE-Cy7 (25-9668-82, Invitrogen, Carlsbad, CA, USA), anti-F4/80 PE (12-4801-80, Invitrogen, Carlsbad, CA, USA) for 30 min at 4 °C and washed for FACS (fluorescence-activated cell sorting) analysis with Guava easyCyte 8HT (Merck Millipore, Burlington, MA, USA).

### Isolation of bone marrow and BALF neutrophils

Bone marrow (BM) was isolated from femurs and tibias by flushing with DMEM. Red blood cells were lysed and the remaining cells were isolated by density gradient centrifugation using discontinuous gradient Percoll (75%, 65%, and 55%, 17089101, Cytiva, Washington DC, USA). Neutrophils were obtained from the 75%−65% interface. BALF neutrophils were separated by anti-Ly6G microbead kit (130-092-332, Miltenyi Biotec, Köln, Germany) according to manufacturer instructions. BALF cells were incubated with anti-Ly-6G-Biotin for 15 min at 4 °C and streptavidin micro-beads for 10 min at 4 °C. Cells were then magnetically sorted by MS columns.

### Measurement of neutrophil ROS

ROS was detected by isoluminol bioluminescence as described previously [27]. BM neutrophils were primed with 100 ng/mL LPS for 3 h and resuspended in Krebs-Ringer phosphate buffer (KRG) containing isoluminol (A8264, Sigma-Aldrich, Darmstadt, Germany) and horseradish peroxidase (P8375, Sigma-Aldrich, Darmstadt, Germany). After equilibration at 37 °C for 5 min, GPR84 agonist 6-OAU was added and the light emission was continuously recorded by Envision 2101 multiplate reader (PerkinElmer, Waltham, MA, USA). To detect the antagonist effect of BGT-004, LPS-primed neutrophils were pre-incubated with BGT-004 for 30 min before the addition of 6-OAU.

### Neutrophil chemotaxis

BM Neutrophils were collected into RPMI-1640 containing 0.25% BSA and 20 mM HEPES at a density of 1 × 10^7^ cells/mL. These cell suspensions (100 μL) were added to a Transwell cup (filter pore size: 3 μm) inserted into a well of 24-well plate (3415, CORNING, Corning, NY, USA) containing 300 μL of buffer with vehicle or GPR84 agonist (10 μM 6-OAU). For the blocking assay, BGT-004 at various concentrations were supplied in both the upper and lower chambers, and GPR84 agonist (10 μM 6-OAU) was added into the lower chamber. Following incubation at 37 °C for 1.5 h, migrated cells were collected from the lower chamber, and the number of cells was counted with Guava easyCyte 8HT (Merck Millipore, Burlington, MA, USA).

### Neutrophil degranulation

Neutrophils were pre-incubated with 20 ng/mL TNFα (1029-TA-050, R&D Systems, Minneapolis, MN, USA) for 30 min and then 10 μM cytochalasin B (CB, HY-16928, MCE, Shanghai, China) for 5 min at 37 °C and stimulated with 6-OAU of various concentrations for 15 min at 37 °C. Then the reaction was stopped with ice-cold PBS buffer and cells were incubated at 4 °C for 30 min with anti-CD63 APC (143906, Biolegend, San Diego, CA, USA). The level of CD63 on the cell surface was analyzed by flow cytometry.

### Western blot

Neutrophils were lysed in RIPA buffer supplemented with a protease inhibitor cocktail (11697498001, Roche, Basel, Switzerland), 10 mM NaF (S7920, Sigma-Aldrich, Darmstadt, Germany) and 1 mM Na_3_VO_4_ (S6508, Sigma-Aldrich, Darmstadt, Germany). Total protein was quantified using the BCA Protein Assay Kit (23225, Thermo Fisher, Cambridge, MA, USA). Samples were mixed with SDS buffer and boiled at 100 °C for 10 min. Cell lysates were loaded on 10% SDS-PAGE and transferred to polyvinylidene difluoride membranes. Non-specific binding was blocked with 5% BSA in TBST buffer for 1 h at room temperature and the membranes were incubated overnight at 4 °C with primary antibody. Then membranes were incubated with appropriate secondary antibodies for 1 h at room temperature. Immunostaining was visualized using Amersham ECL Plus Western Blotting detection reagents (SQ202, Yamei, Shanghai, China) and ChemiDoc imaging system (Bio-Rad, Hercules, CA, USA).

### Immunofluorescence and confocal microscopy

Neutrophils were primed with 100 ng/mL LPS for 3 h and stimulated with 6-OAU. Cells were fixed in 4% paraformaldehyde (PFA) and stained with Wheat embryo agglutinin (WGA, W834, Invitrogen, Carlsbad, CA, USA) for 30 min at room temperature. Then neutrophils were permeabilized in 1% Triton X-100 and blocked using 5% BSA. Primary antibodies of p47^PHOX^ (A1148, Abclonal, Wuhan, China) were added and incubated at 4 °C overnight. Secondary antibodies were stained at room temperature for 1 h. Hoechst (H3570, Invitrogen, Carlsbad, CA, USA) was used for nuclei counterstaining. Samples were imaged through a TCS-SP8 STED confocal microscope (Leica, Wizla, Germany).

### Reverse-transcription and real-time PCR

Total RNA was extracted from cells or mouse tissues using TRIzol reagent (15596026, Invitrogen, Carlsbad, CA, USA) and RNA was reversed to cDNA using a PrimeScript^TM^ RT reagent kit (RR037A, TaKaRa Bio, Shiga, Japan). Real-time PCR was performed using Hieff Qpcr SYBR Green Master Mix (11199ES03, Yeasen, Shanghai, China) and analyzed with a Stratagene Mx 3000 P thermal cycler (Agilent, Santa Clara, CA, USA). Primer sequences were provided in Supplementary Table S1.

### Statistical analysis

Data were analyzed with GraphPad Prism software (GraphPad Software, San Diego, CA, USA) and presented as the means ± SEM. Nonlinear regression analyses were performed to generate dose–response curves and calculate EC_50_ or IC_50_ values. Comparisons between two groups were analyzed by two-tailed Student’s *t* test. The *P* values < 0.05 were considered statistically significant.

## Results

### GPR84 deficiency ameliorates LPS-induced ALI in mice

**Fig. 1:**
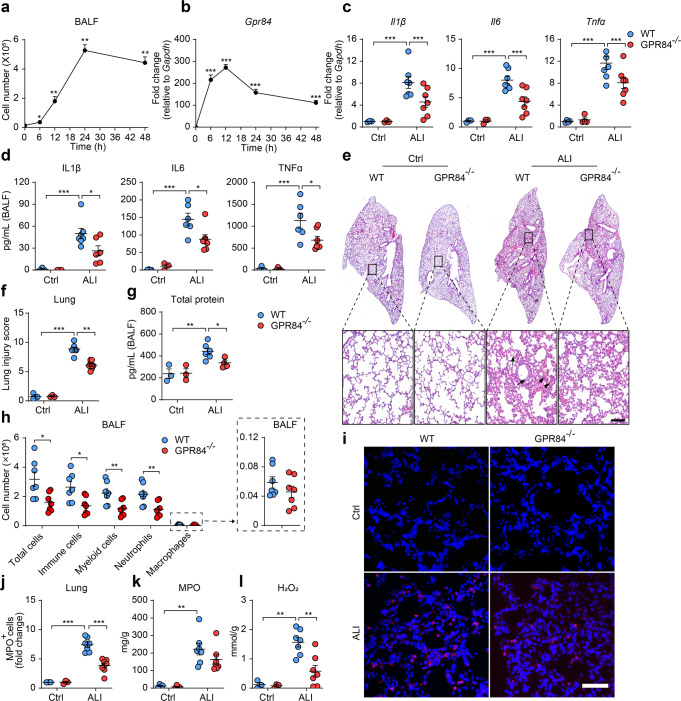
LPS-induced ALI is ameliorated in GPR84^–/–^ mice. The numbers of cells in the BALF (**a**) and Real-time qPCR analysis of GPR84 mRNA levels in BALF cells (**b**) at 0, 6, 12, 24 and 48 h after LPS treatment, *P < 0.05, **P < 0.01, ***P < 0.001 versus 0 h. **c** Real-time qPCR analysis of IL1β, IL6, and TNFα mRNA levels in the lungs of WT and GPR84^–/–^ mice treated with LPS for 24 h. Gene expressions were normalized to GAPDH in the same sample and then normalized to WT control. **d** The concentrations of IL1β, IL6 and TNFα in BALF of WT and GPR84^–/–^ mice treated with LPS for 24 h. Representative images of H&E stained lung sections (**e**) and statistical analysis (**f**) of histological scores. Solid arrow denotes hemorrhage and solid arrow tips point at alveolar septal thickening. Scale bars = 100 μm. **g** Content of BALF total protein in WT and GPR84^–/–^ mice. **h** The cell numbers per mouse of total cells, CD45^+^ immune cells, CD45^+^ CD11b^+^ myeloid cells, CD45^+^ CD11b^+^ Ly6G^+^ neutrophils and CD45^+^ CD11b^+^ Ly6G^-^ F4/80^+^ macrophages in the BALF of WT and GPR84^-/-^ mice treated with LPS for 24 h^. *^P < 0.05, **P < 0.01, *n* = 8. Representative pictures (**i**) and statistical analysis (**j**) of immunofluorescence staining of MPO^+^ neutrophils (red) in lung sections of ALI mice. Nuclei were stained with Hoechst 33258 (blue). Scale bars = 50 μm. **k**, **l** Concentration of MPO and H_2_O_2_ in the lungs of WT and GPR84^–/–^ ALI mice. All data are presented as means ± SEM. *P < 0.05, **P < 0.01, ***P < 0.001.

Mice ALI model was induced via intratracheal instillation of LPS. BALF (bronchoalveolar lavage fluid) was collected at 0, 6, 12, 24 and 48 h post LPS treatment and the number of infiltrating cells in the BALF was significantly increased in a time-dependent manner (Fig. 1a). Quantitative RT-PCR revealed that the expression of GPR84 in the BALF cells also increased significantly after LPS instillation (Fig. 1b). This result suggests that GPR84 might be associated with the inflammatory process of ALI. ALI was then induced in both the wild type (WT) and GPR84^–/–^ mice with LPS and lung tissues were harvested 24 h later. Compared with WT mice, the mRNA levels of the proinflammatory cytokines, including IL1β, IL6 and TNFα were significantly lower in the lung tissue of GPR84^–/–^ mice (Fig. 1c). Consistently, the protein levels of the proinflammatory cytokines were also significantly decreased in GPR84^–/–^ mice (Fig. 1d). Histological analysis showed that GPR84 deficiency alleviated the damage of alveolar structure, hemorrhage and alveolar septal thickening, with a significant decrease in lung injury scores (Fig. 1e, f). Total protein in the BALF was also reduced in GPR84^–/–^ mice, suggesting a less severe lung injury (Fig. 1g).

FACS analysis of the cells in the BALF demonstrated that the total cells, CD45^+^ cells, CD45^+^ CD11b^+^ myeloid cells and CD45^+^ CD11b^+^ Ly6G^+^ neutrophils were all significantly decreased in GPR84^–/–^ mice challenged with LPS (Fig. 1h), while there was no significant difference in CD45^+^ CD11b^+^ Ly6G^-^ F4/80^+^ macrophages between WT and GPR84^–/–^ mice. Among neutrophil granule proteins, myeloperoxidase (MPO) is commonly used to assess the infiltration of neutrophils in lung tissues [28, 29]. Immunofluorescent staining of the pulmonary sections indicated fewer MPO-positive neutrophils in the lung tissue from GPR84^–/–^ mice (Fig. 1i, j), and a slight reduction of MPO (*P* = 0.1972) protein level in the lung tissue was also detected in GPR84^–/–^ mice (Fig. 1k). Neutrophils have been reported to destruct lung structure by producing ROS and releasing a variety of granule proteins [7, 30]. As the main component of ROS, H_2_O_2_ represents lung oxidative stress level [9, 31]. We also observed a significant reduction of H_2_O_2_ in the lungs of GPR84^–/–^ mice (Fig. 1l). These observations clearly suggest that GPR84 participates in ALI pathogenesis.

### GPR84 antagonist attenuates the severity of LPS-induced ALI in mice

**Fig. 2:**
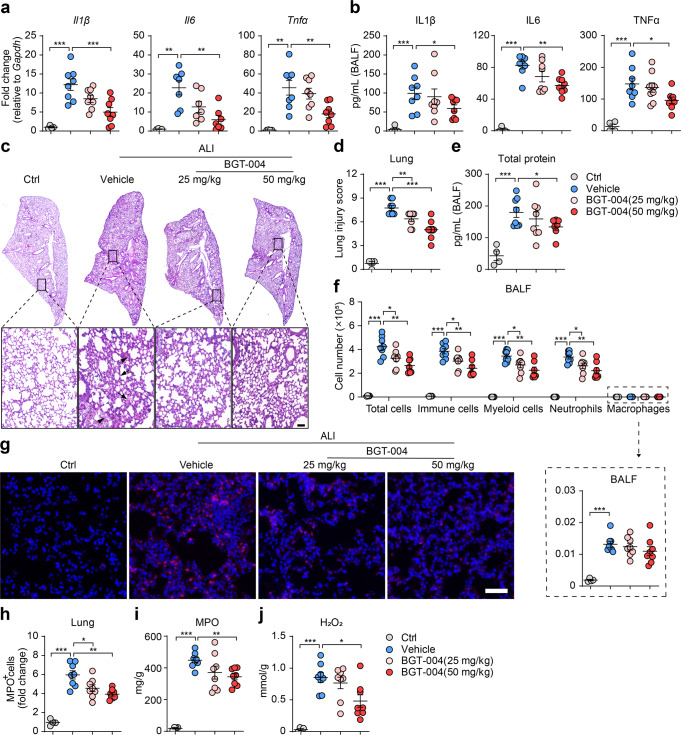
GPR84 antagonist BGT-004 alleviates LPS-induced ALI. ALI mice were orally administered vehicle or BGT-004 (25 and 50 mg/kg) 0.5 h before induction and gavaged 12 h later. Mice were sacrificed 24 h after LPS treatment for following analysis. **a** Real-time qPCR analysis of IL1β, IL6, and TNFα mRNA levels in lung tissues. Gene expressions were normalized to GAPDH in the same sample and then normalized to WT control. **b** IL1β, IL6 and TNFα concentrations in the BALF. Representative pictures of H&E staining of the lung sections (**c**) and statistical analysis (**d**) of the histological scores. Solid arrow indicates hemorrhage and solid arrow tips denote septal thickening. Scale bars = 100 μm. **e** Content of total protein in the BALF. **f** The cell numbers per mouse of total cells, CD45^+^ immune cells, CD45^+^ CD11b^+^ myeloid cells, CD45^+^ CD11b^+^ Ly6G^+^ neutrophils and CD45^+^ CD11b^+^ Ly6G^-^ F4/80^+^ macrophages in BALF, *n* = 8. Immunofluorescence staining (**g**) and statistical analysis (**h**) of MPO^+^ neutrophils (red) in lung sections and nuclei were stained with Hoechst 33258 (blue). Scale bars = 50 μm. The levels of MPO (**i**) and H_2_O_2_ (**j**) in lung tissue. All data are presented as means ± SEM, *P < 0.05, **P < 0.01, ***P < 0.001.

Deletion of GPR84 remarkably ameliorated LPS-induced lung inflammation, we wondered whether blocking GPR84 with small molecule antagonist could provide similar protection. BGT-004 (compound **33**) [24] is a newly discovered GPR84 antagonist, which blocks 6-OAU (1 μM) induced GPR84 activation with an IC_50_ of 8.95 nM. Half an hour before the LPS instillation, mice were orally administered BGT-004 and lung tissues and BALF were collected 24 h after ALI induction. Compared with the vehicle, BGT-004 significantly reduced the mRNA levels of proinflammatory cytokines including IL1β, TNFα and IL6 in the lung tissue at the dose of 50 mg/kg, while a slight reduction was also observed at the dose of 25 mg/kg (Fig. 2a). The protein levels of the proinflammatory cytokines in BALF were also significantly reduced by BGT-004 treatment (Fig. 2b). Histological assessment of lung sections showed that BGT-004 inhibited the infiltration of immune cells into lung parenchyma, ameliorated hemorrhage and prevented septa thickening (Fig. 2c, d). BGT-004 (50 mg/kg) also significantly reduced the total protein level in the BALF (Fig. 2e). FACS analysis revealed that BGT-004 reduced the number of total cells, CD45^+^ cells, CD45^+^ CD11b^+^ myeloid cells and CD45^+^ CD11b^+^ Ly6G^+^ neutrophils in a dose-dependent manner (Fig. 2f). Both the number of MPO-positive neutrophils and the protein level of MPO in lung tissue were significantly reduced in BGT-004-treated mice (Fig. 2g–i). And the concentration of H_2_O_2_ was also reduced by BGT-004 treatment (Fig. 2j). These data suggest that blocking GPR84 with a small molecule antagonist protects mice from ALI.

### GPR84 regulates neutrophil chemotaxis, ROS production and degranulation

**Fig. 3:**
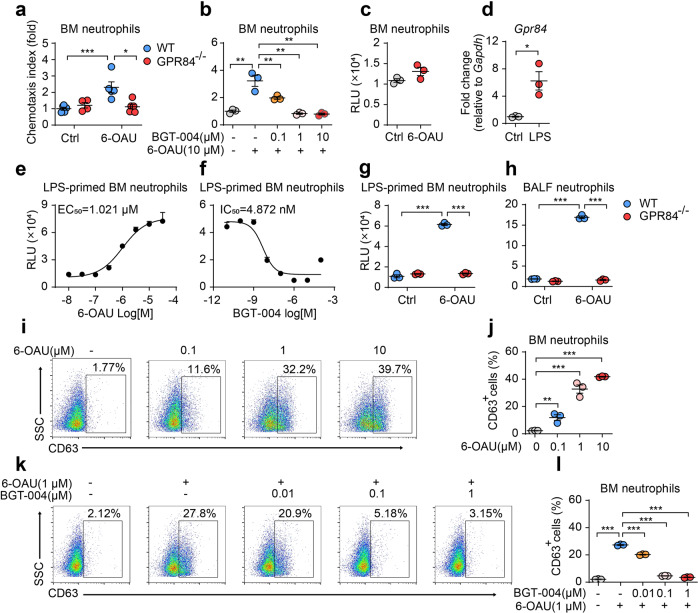
Activation of GPR84 promotes neutrophil chemotaxis, ROS production and degranulation. **a** Chemotaxis of WT and GPR84^–/–^ BM neutrophils in response to 6-OAU (10 μM). **b** Chemotaxis of BM neutrophils toward 6-OAU (10 μM) in the presence of BGT-004 (0.1, 1 or 10 μM). The chemotaxis index was assessed by counting cells in the lower chamber of the transwell and then normalized to control of the WT group. **c** ROS signal of unprimed WT BM neutrophils stimulated with 6-OAU (3 μM). **d** mRNA levels of GPR84 in BM neutrophils primed with LPS for 3 h. Gene expressions were normalized to GAPDH in the same sample and then normalized to control. **e** Dose-dependent stimulation of ROS release from LPS-primed WT BM neutrophils by 6-OAU. **f** Dose-dependent inhibition of 6-OAU (3 μM) stimulated ROS release from LPS-primed WT BM neutrophils by BGT-004. ROS production of LPS-primed WT and GPR84^–/–^ BM neutrophils (**g**) or BALF neutrophils from ALI mice (**h**) after 6-OAU (10 μM) stimulation. Representative FACS plots (**i**) and statistical analysis (**j**) of CD63^+^ BM neutrophils after 6-OAU stimulation. Representative FACS plots (**k**) and statistical analysis (**l**) of CD63^+^ cells in BM neutrophils stimulated with 6-OAU (1 μM) in the presence of various concentrations of BGT-004. All data are presented as means ± SEM, *P < 0.05, **P < 0.01, ***P < 0.001.

The in vivo experiments revealed that deleting or blocking GPR84 reduces the pathogenesis of ALI in mice, possibly by regulating neutrophil functions. We then explored the roles of GPR84 in neutrophils in vitro. The GPR84 agonist 6-OAU (10 μM) could induce significant chemotaxis of the BM (bone marrow) neutrophils isolated from WT mice in the transwell assay, while such effect diminished in cells isolated from GPR84^–/–^ animals (Fig. 3a). The GPR84 antagonist BGT-004 was also found to block 6-OAU-induced neutrophil chemotaxis in a dose-dependent way (Fig. 3b). In the ROS production assay, 6-OAU could only stimulate a weak ROS signal in BM neutrophils without priming (Fig. 3c). Considering that neutrophils were activated by a pro-inflammatory microenvironment in mice ALI model, we stimulated BM neutrophils with LPS for 3 h and found a significant upregulation of GPR84 (Fig. 3d). In LPS-primed BM neutrophils, 6-OAU could stimulate a massive ROS signal (EC_50_ = 1.021 μM), which was dampened by BGT-004 with an IC_50_ of 4.872 nM (Fig. 3e, f). In contrast, 6-OAU could not stimulate ROS production from LPS-primed GPR84^–/–^ BM neutrophils (Fig. 3g). Neutrophils isolated from the BALF of WT ALI mice also exhibit high ROS signal in the presence of 6-OAU, while the GPR84^–/–^ BALF neutrophils did not respond to 6-OAU stimulation (Fig. 3h). Activation of neutrophils not only result in the production of ROS but also the release of cytosolic granules known as degranulation [32]. Neutrophil degranulation requires a prime process for kinase phosphorylation and cytoskeleton redistribution [33]. The BM neutrophils were primed with TNFα for 30 min and then incubated with cytochalasin B (CB) for 5 min before 6-OAU stimulation, and the degranulation process was determined by measuring the membrane CD63 level [34]. FACS analysis revealed that the cell surface CD63 was significantly upregulated by 6-OAU (Fig. 3i, j), while such effect could be blocked by BGT-004 in a dose-dependent manner (Fig. 3k, l). Taken together, GPR84 plays important roles in neutrophil functions including migration, ROS production and degranulation.

### GPR84 modulates neutrophils ROS production via Lyn-AKT/ERK pathway

**Fig. 4:**
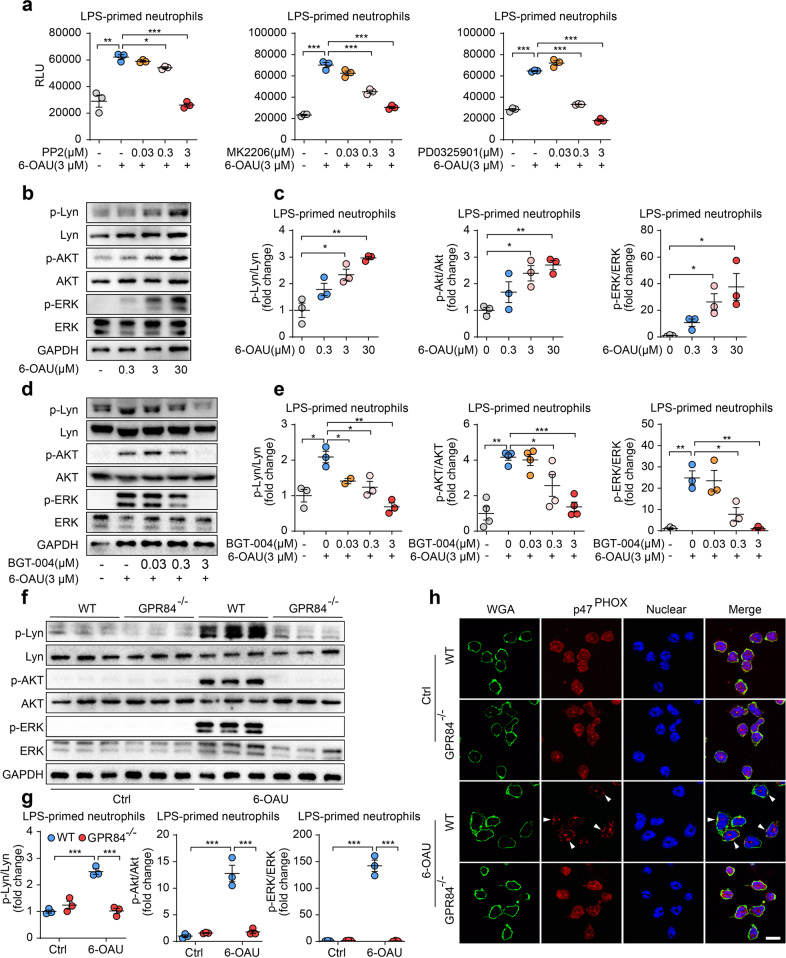
GPR84 regulates ROS production via Lyn, AKT and ERK1/2 phosphorylation. BM neutrophils were used for following experiments.**a** 6-OAU (3 μM)-induced ROS production in LPS-primed neutrophils in the presence of various concentrations of Lyn inhibitor PP2, AKT inhibitor MK2206 and ERK1/2 inhibitor PD0325901. Representative Western blot (**b**) and statistical analysis (**c**) of Lyn, AKT and ERK1/2 phosphorylation in LPS-primed neutrophils stimulated with 6-OAU (0.3, 3 or 30 μM). Representative Western blot (**d**) and statistical analysis (**e**) of Lyn, AKT and ERK1/2 phosphorylation stimulated by 6-OAU (3 μM) in the presence of BGT-004 in LPS-primed neutrophils. Representative Western blot analysis (**f**) and quantitation (**g**) of Lyn, AKT and ERK1/2 phosphorylation in LPS-primed WT and GPR84^–/–^ BM neutrophils treated with 6-OAU (3 μM). All blots were representative results of three independent treatments and densitometry analysis was performed as the ratio between the phosphoprotein versus the same total protein and then normalized to control. **h** Representative immunofluorescence staining of cell membrane (WGA-labeled, green), p47^PHOX^ (red) and nuclei (blue) in LPS-primed WT and GPR84^-/-^ neutrophils stimulated with 6-OAU (3 μM). Scale bars = 10 μm. All data are presented as means ± SEM, *P < 0.05, **P < 0.01, ***P < 0.001.

Previous reports have demonstrated that SFKs (Src family kinases) play critical roles in neutrophil activation [35]. Lyn (Lck/Yes-related novel tyrosine kinase) is a member of the SFKs family highly expressed in neutrophils and is suggested to mediate ROS production. In intracellular signal transduction, Lyn can activate both AKT and ERK1/2, either of which can phosphorylate p47^PHOX^ [14, 36], a key subunit of NADPH oxidase. To investigate whether the Lyn-AKT/ERK pathway participates in GPR84-mediated neutrophil ROS production, several small molecule inhibitors were tested. Inhibition of Lyn by PP2, AKT by MK2206 or ERK1/2 by U0126 all significantly reduced 6-OAU-induced ROS production (Fig. 4a). Western blot analysis showed that 6-OAU could induce phosphorylation of Lyn, AKT and ERK1/2 in a concentration-dependent manner in LPS-primed BM neutrophils (Fig. 4b, c), which could be blocked by GPR84 antagonist BGT-004 (Fig. 4d, e). In LPS-primed GPR84^-/-^ BM neutrophils, no phosphorylation signals of Lyn, AKT and ERK1/2 were detected by 6-OAU stimulation (Fig. 4f, g). NADPH oxidase, which is crucial for the production of ROS, is composed of four cytosolic subunits and two membrane-bound components. Among the cytosolic proteins, p47^PHOX^ is an adaptor subunit essential for the assemble of NADPH oxidase [37]. Previous studies suggested that p47^PHOX^ could translocate to the membrane after phosphorylation by ERK1/2 and AKT [38, 39]. In LPS-primed neutrophils, 6-OAU stimulation could induce the translocation of p47^PHOX^ to the plasma membrane, while this effect could not be observed in GPR84^–/–^ neutrophils (Fig. 4h). Taken together, our results indicate that GPR84 regulates ROS production from neutrophils via the Lyn-AKT/ERK pathway.

### GPR84 deficiency or inhibition attenuates BLM-induced lung inflammation

**Fig. 5:**
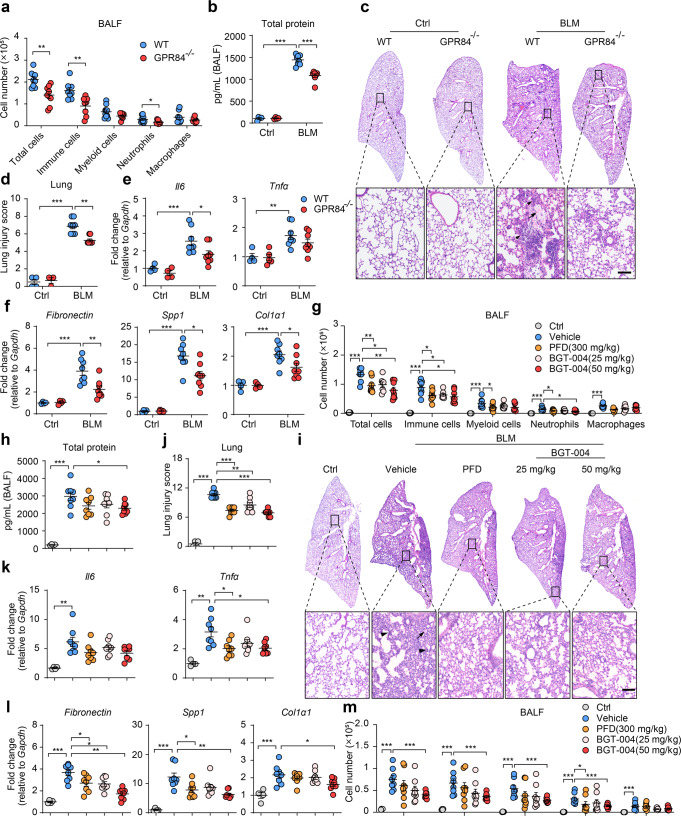
Knockout or blockade of GPR84 reduces BLM-induced lung injury. **a**–**f** Lung injury was induced in WT and GPR84^-/-^ mice with intratracheal instillation of 2 U/kg BLM and the animals were sacrificed 7 days later for following analysis. **a** The cell numbers per mouse of total cells, CD45^+^ immune cells, CD45^+^CD11b^+^ myeloid cells, CD45^+^ CD11b^+^ Ly6G^+^ neutrophils and CD45^+^ CD11b^+^ Ly6G^-^ F4/80^+^ macrophages in the BALF. **b** Total protein levels in the BALF. Representative images of H&E staining of the lung sections (**c**) and the statistical analysis of the histological scores (**d**). Solid arrows denote immune cells infiltration and solid arrow tips shows the hyaline accumulation. Scale bars = 100 μm. mRNA levels of proinflammatory cytokines IL6 and TNFα (**e**) and profibrotic factors Fibronectin, Spp1 and Col1α (**f**) in the lung tissues. Gene expressions were normalized to GAPDH in the same sample and then normalized to WT control. **g**–**l** BLM-treated mice were gavaged with vehicle, PFD (300 mg/kg, bid) or BGT-004 (25 and 50 mg/kg, bid) and sacrificed 7 days after BLM treatment for further analysis. **g** FACS analysis of the cell numbers per mouse of the total cells, CD45^+^ immune cells, CD45^+^CD11b^+^ myeloid cells, CD45^+^ CD11b^+^ Ly6G^+^ neutrophils and CD45^+^ CD11b^+^ Ly6G^-^ F4/80^+^ macrophages in the BALF. **h** Total protein levels in the BALF. Representative images of H&E staining of the lung sections (**i**) and the statistical analysis of the histological scores (**j**). Solid arrow tips indicate the hyaline formation and solid arrows point at immune cells accumulation. Scale bars = 100 μm. mRNA levels of proinflammatory cytokines (**k**) and profibrotic factors (**l**) in the lung tissues. Gene expressions were normalized to GAPDH in the same sample and then normalized to control. (**m**) Mice were orally administered vehicle, PFD (300 mg/kg, bid) or BGT-004 (25 and 50 mg/kg, bid) 0.5 h before BLM instillation and gavaged again 12 h later. BALF was collected 24 h after BLM treatment. Cell numbers per mouse of the total cells, CD45^+^ immune cells, CD45^+^CD11b^+^ myeloid cells, CD45^+^ CD11b^+^ Ly6G^+^ neutrophils and CD45^+^ CD11b^+^ Ly6G^-^ F4/80^+^ macrophages were analyzed by FACS. All data are presented as means ± SEM, *P < 0.05, **P < 0.01, ***P < 0.001.

Neutrophils also play critical roles in BLM (bleomycin)-induced lung inflammation [40, 41]. We then induced lung injury in GPR84^–/–^ mice by intratracheal instillation of BLM. BALF were collected on Day 7 after induction, the numbers of total cells, CD45^+^ cells and CD45^+^ CD11b^+^ Ly6G^+^ neutrophils were significantly reduced in GPR84^–/–^ mice (Fig. 5a). Total protein in the BALF was also significantly reduced in GPR84^–/–^ mice (Fig. 5b). H&E staining of the lung sections indicated that deletion of GPR84 decreased lung injury with reduced hyaline formation, preserved alveolar structure, and less accumulation of infiltrated cells (Fig. 5c, d). In lung tissues, the upregulation of proinflammatory cytokines (TNFα and IL6) and pro-fibrotic genes (Fibronectin, SPP1 and Col1α) were significantly lower in GPR84^–/–^ mice (Fig. 5e, f). We also tested whether GPR84 antagonist BGT-004 had any effect on BLM-induced inflammation. BGT-004 were gavaged at 25 or 50 mg/kg (b.i.d) since BLM induction and the mice were sacrificed 7 days later. BGT-004 at 50 mg/kg significantly reduced the numbers of total cells, CD45^+^ immune cells and neutrophils in the BALF (Fig. 5g). BALF total protein was also reduced by BGT-004 treatment (Fig. 5h). Histological analysis of lung tissue demonstrated that BGT-004 reduced alveolar collapse, hyline accumulation and cell infiltration compared to vehicle (Fig. 5i, j). Proinflammatory cytokines TNFα and pro-fibrotic genes in lung tissue were downregulated by 50 mg/kg BGT-004 (Fig. 5k, l), the reducing effect was similar to the PFD (pirfenidone, 300 mg/kg, b.i.d), a drug used to treat lung fibrosis. Neutrophils are believed to be the first immune cells reaching the injury site and play a central role in initiating acute inflammatory responses which may lead to lung injury [42]. We also tested the effect of BGT-004 at the early stage of BLM-induced inflammation. BGT-004 (25 or 50 mg/kg) or PFD (300 mg/kg) was orally delivered 0.5 h before BLM-induction and gavaged again 12 h later. Mice were sacrificed 24 h after BLM instillation and BALF cells were collected for analysis. We found that BGT-004 at 50 mg/kg significantly reduced neutrophils infiltration in the acute phase of inflammation (Fig. 5m). Taken together, our results demonstrate that in BLM-induced lung inflammation, deletion or inhibition of GPR84 also provides protective effects.

## Disscusion

Neutrophils play critical role in ALI development, the number of neutrophils in the BALF of ALI patients is tens of times higher than that of healthy controls [43]. Neutrophils isolated from the BALF of ALI patients acquire stronger chemotaxis and ROS-releasing ability. Increased levels of neutrophil-derived MPO and elastase were also observed in ALI patients. Here we demonstrate that GPR84 regulates neutrophil chemotaxis and ROS production, deleting or blocking GPR84 could reduce neutrophil infiltration and ROS release, and provide protective effect in ALI animal models.

ROS is a powerful weapon of neutrophils to fight against microorganisms, but it is also toxic to host cells when accumulated excessively and contributes to ALI progression [44]. ROS directly evokes cell injury by inducing membrane lipid oxidation and DNA lesion. These oxidants also promote inflammatory cytokines expression by activating NF-κB pathway [8]. Some antioxidants contribute to protecting the endothelium from oxidant injury and have an essential role in tissue repair. Antioxidants such as Vitamin C (Vc) [45, 46] and quercetin [47] are very effective scavengers of ROS and can be used to protect against ROS-induced tissue damage. As a matter of fact, both have been used to treat COVID-19 induced ALI. Intravenous Vc not only reduced IL-6 level, increased blood oxygen content, but also reduced mortality rate in COVID-19-associated ALI [46]. Another clinical trial demonstrated that herbs with a high quercetin content also provided significant benefits in reducing immune response and hospitalization days in COVID-19-caused ALI patients [48].

**Fig. 6:**
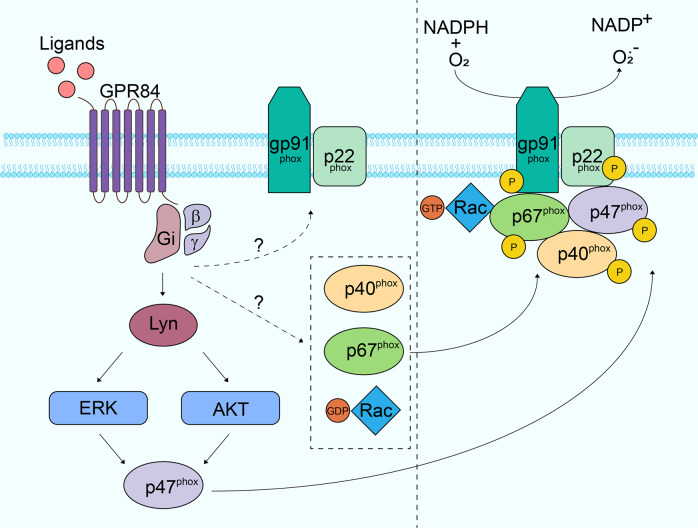
Mechanism of GPR84-induced ROS production in neutrophils. Activation of GPR84 by its ligands induces the activation of Lyn. Activated Lyn phosphorylates the downstream AKT and ERK1/2. These two kinases then phosphorylate and induce membrane translocation of p47^PHOX^, a critical step in forming the functional NADPH oxidase. Whether and how GPR84 regulates the other subunits of the NADPH oxidase remain to be elucidated.

To block ROS production is another way to reduce ROS content. Neutrophils produce ROS mainly via NOX2. NOX2 consists of four cytosolic subunits, including p67^PHOX^, p47^PHOX^, p40^PHOX^ and Rac2, and two membrane-bound proteins named gp91^PHOX^ and p22^PHOX^ [37]. During ROS production, the segregated components assemble at the membrane and transfer electrons from NADPH to oxygen [37]. The assembly of the NOX2 complex requires phosphorylation of the subunits at specific residues and activation of Rac2. A variety of molecules mediate the prime and activation of NOX2 [49]. During immune response, signals from Toll-like receptors (TLRs) and cytokines receptors such as TNFα prime neutrophils to achieve more robust activation of NOX2 through mitogen-activated protein kinases (MAPK), including p38 and ERK1/2 [33]. The Fc receptors (FcR), which capture the antibody-opsonized pathogens, trigger p40^PHOX^ activation and ROS production via Syk-PI3K pathway or SFKs (Lyn, Hck and Fgr) [50, 51]. Bacterial metabolites such as formyl-methyl-leucine-phenylalanine (fMLP) can activate FPR1, which leads to phosphorylation of SFKs and downstream Vav-Rac and the Akt/ERK pathways, and stimulate p47^PHOX^ translocation and ROS production [14]. FPR1 can also activate the PLCβ and PI3Kγ signaling cascades, leading to the activation of PKC, which also regulates ROS production [52, 53]. Some of the above pathways also participate in ROS production stimulated by other GPCRs. Leukotriene B4 receptor-mediated ROS generation was blocked by ERK inhibitor, and C5aR-induced superoxide was also reduced in neutrophils without MAPK-activated protein kinase 2 [54, 55]. Here we discover that GPR84 activation induces Lyn, AKT and ERK1/2 phosphorylation, which in turn stimulates the phosphorylation and membrane translocation of the p47^PHOX^. Blocking GPR84 or these pathways with inhibitors could all block ROS production (Fig. 6).

An interesting phenomenon we observed was that GPR84 agonist could only induce small amount of ROS production in mouse BM neutrophils, but could induce large amount of ROS in BALF neutrophils isolated from ALI mice. This is largely due to the activation of neutrophils in the BALF of ALI mice. In vitro priming of the BM neutrophils with LPS significantly increased the expression of GPR84, and also greatly enhanced the ROS production after GPR84 activation. Previous reports have suggested that LPS stimulation could also upregulate gp91^PHOX^, and induced partially phosphorylation and the translocation of p47^PHOX^ [56, 57]. Thus LPS-primed neutrophils not only express higher levels of GPR84, but also higher levels of the ROS production machinery.

MPO is another enzyme to produce oxidative molecules such as hypochlorous acid, it is mainly released by neutrophils via degranulation [28]. Upregulation of MPO in the BALF is also a hallmark of ALI [43]. A number of GPCRs such as FPR1 and C5aR have been reported to mediate neutrophil degranulation [58, 59]. Previous studies showed that SFKs (Lyn, Hck, and Lyn) and Rac not only play a role in NOX2 activation but also influence neutrophil degranulation [60, 61]. We found activation of GPR84 induces neutrophils degranulation, possibly also via the Lyn-Rac pathway.

In summary, we demonstrated that GPR84 plays an important role in lung inflammation. Deletion or chemical blockade of GPR84 alleviates LPS- or BLM-induced lung inflammation in mice. Further studies show that activation of GPR84 promotes neutrophil infiltration, ROS production and degranulation. These results suggest that targeting GPR84 on neutrophils might be a potential therapy for the treatment of ALI.

## Supplementary information

Supplementary Information
