# Supplementary material for: GPR84 regulates pulmonary inflammation by modulating neutrophil functions
Source: Acta Pharmacol Sin. 2023 Apr 4;44(8):1665–75. doi: 10.1038/s41401-023-01080-z (PMC10072043; doi:10.1038/s41401-023-01080-z)
Supplement: Supplementary file 1 — Supplementary Information [file 41401_2023_1080_MOESM1_ESM.docx]

**Supplementary Information**

**Supplementary Table 1. Oligonucleotides used for Real-time PCR analysis**

| **Gene** | **Forward primer (5'-3')** | **Reverse primer (5'-3')** |
| --- | --- | --- |
| GPR84 | CTCCTGCTACCATGAGTCTGT | GTGCAGTAGAGTAGATCAGCCA |
| TNFα | CTGAACTTCGGGGTGATCGG | GGCTTGTCACTCGAATTTTGAGA |
| IL-1β | GCAACTGTTCCTGAACTCAACT | ATCTTTTGGGGTCCGTCAACT |
| IL-6 | TAGTCCTTCCTACCCCAATTTCC | TTGGTCCTTAGCCACTCCTTC |
| Col1α | GCCAAGAAGACATCCCTGAA | GGCAGAAAGCACAGCACTC |
| Fibronectin | TCTGGGAAATGGAAAAGGGGAA | CACTGAAGCAGGTTTCCTCGGTT |
| Spp1 | GGAGGAAACCAGCCAAGG | TGCCAGAATCAGTCACTTTCAC |
| GAPDH | TCAACAGCAACTCCCACTCTT | ACCCTGTTGGTGTAGCCGTAT |
